# Spinal Anaesthesia for Caesarean Section in Pregnant Women with Fetal Distress: Time for Reappraisal

**Published:** 2014-06

**Authors:** J. M. Afolayan, T. O. Olajumoke, S. E. Esangbedo, N. P. Edomwonyi

**Affiliations:** 1 Department of Anaesthesia, Ekiti State University Teaching Hospital, Ado-Ekiti, Nigeria;; 2 Department of Anaesthesia, LAUTECH Teaching Hospital, Osogbo, Nigeria;; 3 Department of Anaesthesia, University of Benin Teaching Hospital, Benin City, Nigeria

**Keywords:** Emergency caesarean section, fetal distress, spinal anaesthesia

## Abstract

Residents’ competency-based training and multidisciplinary cooperation are needed for rapid sequence spinal anaesthesia for fetal distress. Multiple standard but ‘crash’ spinal anaesthesia for non-obstetric procedures is imperative for acquisition of experienced hands. The purpose of this review is to share our modest experiences in the use of rapid spinal anaesthesia for emergency Caesarean delivery in pregnant women complicated with fetal distress. Fetal distress diagnosis is made promtly, intravenous line put in place in labour ward. Pre-loading or not, one-touch, non-touch spinal technique prevents unnecessary delay and further fetal hypoxic injury. Spinal pack is on stand by in the operating room at all time. Preloading is possible during the waiting period for other care providers otherwise coloading is used. A single wipe of the back with chlorhexidine lotion is frequently used for scrubbing. Lidocaine infiltration or spay is essential and does not waste time but opioid as adjuvant to bupivacaine wastes a lot of time to constitute and measure. So, opioid should be avoided. Average of 2.5 ml of 0.5% hyperbaric bupivacaine is frequently used in our centres. Surgery starts almost immediately after cleaning and drapping of the patient by the obstetrician. Ephedrine is made handy and constituted in case there is hypotension which fluid alone cannot treat.

## INTRODUCTION

Progressive breakthrough in obstetric anaesthesia, triumph over complications of regional anaesthesia and the thrilling conquest of unnecessary delay in the establishment of subarachnoid block have been the tripod on which experienced anaesthetist stands in his quest to induce rapid sequence spinal anaesthesia for Caesarean delivery among pregnant women with fetal distress (1). Presently, with high level of multidisciplinary communication and cooperation, rapid spinal anaesthesia for Caesarean delivery due to fetal distress is no longer controversial and does not pose any dilemma for the world anaesthetists especially the ones in the developing world such as Nigeria (1-3). Evolution of spinal anaesthesia with its revolutionary trend in the areas of anatomy, physiology, pathology and pharmacology has reduced to the bearest minimum the incidence of general anaesthesia-related difficult intubation, aspiration pneumonitis, hypoxia and unconscious mother in the presence of awake neonate (4, 5).

Spinal anaesthesia can be performed faster now than before among residents at University of Benin Teaching Hospital, Nigeria therefore it is becoming a more acceptable option for pregnancy complicated with fetal distress. Despite an increasing use of spinal anaesthersia for fetal distress in this hospital, morbidity and mortality following its use are not so common. Rarity of these complications and rapidity by which the block is induced are as a result of cardinal elements of residents’competency-based training in spinal anaesthesia not only for fetal distress but all other simulated rapid spinal anaesthesia for non-obstetric population. Based on our personal experiences and evidence from other scientific literature, there is need for us to re-inforce the usefulness of spinal anaesthesia in obstetric emergency while popularising rapid spinal anaesthesia for fetal distress.

## DISCUSSION

Fetal distress is the term used to describe fetal asphyxia (fetus with compromised gaseous exchange in the antepartum or intrapartum period). The fetal hypoxia resulting from this compromise may result in permanent fetal damage or death if not reversed or if the delivery is unnecessarily delayed (6). Fetal distress is in category 1 Caesarean section because there is an immediate threat to life of the fetus. Categorisation of urgency of Caesarean section is shown in Table 1.

**Table 1:** Categorisation of urgency of Caesarean Section (3)

Grade	Definition (at time of decision to operate)

Category 1	Immediate threat to life of woman or fetus
Category 2	Maternal or fetal compromise, not immediately life-threatening
Category 3	Needing early delivery but no maternal or fetal compromise
Category 4	At a time to suit the woman and maternal team.

Following hypoxaemia, increased fetal blood pressure occurs due to constriction of peripheral vessels which results in slowing of fetal heart rate and respiratory compromise. Moderate hypoxia causes circulating blood to redistribute to the brain, heart and adrenal at the expenses of peripheral organs (lung, skin etc). With prolonged hypoxia, blood flow to the brain stem is maintained and even greater than that in other brain regions. With prolonged and severe hypoxia, glucose is metabolised anaerobically, lactate concentration is increased leading to lactic acidosis (metabolic acidosis). As hypoxia progresses unabated, cerebral metabolism collapses, neuronal membrane depolarizes and voltage-gated calcium channel open with increased influx of calcium ions, these changes result in neuronal death (7).

Normal healthy fetus tolerates transient hypoxia following normal uterine contractions. But conditions such as uteroplacental vascular insufficiency, cord accident (compression, knot, prolapse), reduction in uterine perfusion, placental abruption, fetal infection and reduced fetal reserves can cause reduction in uteroplacental blood flow and persistent hypoxia. In other to prevent fetal ischaemic injury, fetus exhibit compensatory measures which involve redistribution of blood to vital organs (7). This compensatory mechanism enables the fetus to survive asphyxia unless the insult is profound or prolonged. The most common asphyxial stresses imposed on the fetus during labour are insufficiency of uterine blood flow or umbilical blood flow and occasionally decrease in uterine arterial oxygenation (8). Each of these stressors produces characteristic fetal heart rate patterns: late deceleration, variable decelerations or prolonged bradycardia (6). The diagnostic markers of fetal distress are presented in Table 2.

**Table 2:** Diagnostic markers of fetal distress

Pattern	Markers

Reassuring (close monitoring)	• Mild variable deceleration
	• Early deceleration
	• Acceleration
	• Bradycardia (100-120 bpm)
Non-reassuring (resuscitation is needed)	• Tarchycardia
	• Bradycardia (80 to 100 bpm)
	• Saltatory Variability (>25 bpm)
	• Decrease in baseline Variability
Ominous (Immediate delivery)	• Persistent late deceleration with loss of beat to beat variability
	• Persistent severe variable deceleration
	• Acidosis (pH<7.2)
	• Prolonged severe bradycardia (<80 bpm)
	• Persistent tachycardia
	• Loss of variability (flat tracing)
	• Meconium stained liquor

Apart from this, the fetus reduces its oxygen utilization to as low as 50%. Persistent hypoxia results in acidosis and worsens neonatal outcome. A decrease or loss of variability in the presence of this pattern is a sign that the physiologic compensatory mechanism is overwhelmed.

## FETAL HEART RATE PATTERNS

### Electronic Monitoring

Electronic fetal heart rate monitoring is commonly used to access fetal well-being during labour. Although detection of fetal compromise is one of the benefits of fetal monitoring, there are also risks including false positive tests that may result in unnecessary surgical intervention. The fetal heart rate undergoes constant beat-to-beat and minute-to-minute adjustments in response to fetal environment and stimuli (8). Fetal heart rate patterns are classified as normal, reassuring, nonreassuring or ominous.

### Normal Patterns

The American College of Obstetricians and Gynecologists (ACOG) recommends that intermittent auscultation of the fetal heart rate is equivalent to continuous electronic fetal heart monitoring (9). The normal range of fetal heart rate is between 120 and 160 bpm. Fetal bradycardia (<120 bpm) or tachycardia (>160 bpm) may be associated with fetal asphyxia (hypoxia). Apart from hypoxia, persistent fetal bradycardia may be associated with administration of beta-adrenergic blocking drugs, opioids, oxytocin, supine hypotension syndrome, congenital heart block or cholinergic agents. Persistent fetal tachycardia may be associated with fever, beta sympathomimetic drugs, anticholinergic agents or dehydration (10).

### Baseline Variability

Since its first trial in Yale University, in 1958, electronic fetal heart rate (FHR) monitoring has been helpful and widely used in detecting fetal compromise occasioned by the influence of intra-uterine environment. Baseline heart rate is the heart rate that occurs between uterine contractions. Therefore, baseline heart rates are recorded during contraction-free period. Since heart rate ranges between 120 and 160 bpm, baseline FHR can be any where between 120 and 160 bpm (8).

[Autonomic nervous system is responsible for the control of fetal heart rate. The vagus conveys the inhibitory pathway whereas the excitatory one is conveyed by the sympathetic nervous system]. Fetal heart rate variability is the normal irregular variation which produces characteristic jagged or rough appearance of the tracing instead of smooth line. Fetal heart rate variability has become one of the important markers of fetal well-being.

### Short-term (beat-to-beat) Variability

This is a fluctuation in FHR between successive beats. Beat-to-beat variability is the oscillation of the FHR around the baseline in amplitude of 5 to 10 bpm. Clinical evidence suggested that loss of beat-to-beat variability is more significant than loss of long-term variability (11).

### Long-term Variability

This is a fluctuation around the fetal heart rate baseline. It has an amplitude of 6 to 25 bpm.

This is subdivided into three:

- **Decreased Variability:** Minimal variability (0-5 bpm);
- **Moderate Variability:** Normal variability (6-25 bpm);
- **Marked Variability:** Saltatory variability (>25 bpm).

The long-term variability that is desirable for fetus is the moderate type (6-25 bpm) that is, the variation or fluctuation from the baseline should not be less than 6 bpm or greater than 25 bpm. Any variation outside this moderate variation is considered to be abnormal.

### Deceleration

In a normal phenomenon, uterine contractions accelerate fetal heart rate above baseline by at least 15 bpm for at least 15 seconds. Acceleration is different from tachycardia. Tachycardia is heart rate above 160 bpm. Acceleration is increase in heart rate above baseline heart rate. Acceleration is a reassuring sign, but in the face of hypoxia, baroreceptor influence comes into play through the vagus nerve by eliciting selective peripheral vasoconstriction with resultant deceleration. This is subdivided into three.

### Early deceleration

This is caused by parasympathetic stimulation following mild stress of hypoxia during normal uterine contractions. Stress as little as head compression may result in vagal stimulation and manifest deceleration instead of acceleration during uterine contractions. This deceleration begins at the onset of contraction and returns to the baseline rate immediately after uterine contraction. The deceleration is less than 20 bpm.

### Late Deceleration

The heart rate decreases in response to vagal stimulation caused by utero placental insufficiency. This insufficiency is provoked by uterine contractions which further worsens the degree of fetal hypoxia. The deceleration begins 10 to 30 seconds after the beginning of uterine contraction and terminates 10 to 30 seconds after the end of the uterine contraction. There is time lag between uterine contraction and deceleration in late deceleration unlike early deceleration. Persistent late deceleration is injurious to the fetus especially when there is associated loss of short-term variability.

### Variable Deceleration

This deceleration is as a result of umbilical cord compression and the deceleration observed is not due to uterine contractions. Pressure on the cord initially occludes the umbilical vein resulting in acceleration (it indicates a healthy response). The subsequent occlusion of umbilical artery results in deceleration. This deceleration can cause fetal damage if it is persistent.

### Tachycardia

Fetal tachycardia is defined as baseline heart rate greater than 160 bpm. This is considered a non-reassuring pattern of fetal heart rate. Mild tachycardia (160 to 180 bpm), and severe tachycardia (>180 bpm). Tachyarrythmia or congenital anomalies are usually responsible for fetal heart rate greater than 200 bpm.

### Bradycardia

Fetal bradycardia is defined as a baseline heart rate less than 120 bpm. Mild bradycardia (100 to 120 bpm) is common in postdatism, transverse presentations. This is not associated with fetal acidosis provided there is a normal variability. Moderate bradycardia (80 to 100 bpm) is a non-reassuring pattern. Severe bradycardia (<80 bpm) lasting for 3 minutes or more is an ominous sign.

## MANAGEMENT OF FETAL DISTRESS

### Labour ward

As outlined in Table 3, fetal resuscitation should commence right from labour ward. The resuscitation helps to improve placental blood flow and oxygen delivery to the fetus. In the labour ward emergency intravenous access must be put in place for all pregnant women scheduled for caesarean delivery. Sixteen guage cannula is preferable. This has twin benefits of fetal resuscitation by giving the mother intravenous fluid to increase placental blood flow and eliminating delay in placing intravenous cannula in the theatre during rapid sequence induction of spinal anaesthesia for fetal distress.

**Table 3:** Intra-uterine fetal resuscitation

1	Stop oxytocin stimulation	This improves utero placental blood flow and oxygen delivery to the fetus
2	Full lateral position	This prevents supine hypotension syndrome and improves oxygen delivery
3	Oxygen by face masks or nasal prongs	It improves oxygen delivery to the fetus
4	Give intravenous fluid (1 litre crystalloid)	It improves utero placental blood flow and oxygen delivery to the fetus
5	Vasopressor is given (eg ephedrine)	This treats low blood pressure and improves placental blood flow
6	Tocolytic agent is given to stop uterine hyper stimulation	This improves placental blood flow and oxygen delivery

### Decision-Delivery Rule: Is it valid?

A decision to delivery rule of 30minutes is a concept initiated by the American College of Obstetricians and Gynecologists (12). Its relevance to fetal distress is controversial. Decision to delivery time is the time line between a decision being made and the delivery of the baby. Chauhan *et al* (13) compared perinatal outcomes in patients at term in whom the decision-delivery time for caesarean section was due to suspected fetal distress. They concluded that though a caesarean decision-delivery time that is less than or equal to 30minutes is desirable for fetal distress but failure to achieve this goal is not associated with a measurable negative impact on newborn.

Mackenzie *et al* (14) in a similar study observed that fewer than 40% intrapartum deliveries by caesarean section for fetal distress were achieved within 30minutes of the decision. Katz *et al* (15) in his study with more serious level of fetal hypoxia in maternal cardiac arrest (perimortem) concluded that for fetal salvage less than 5minutes decision to delivery is ideal but rarely helpful after 15 minutes. At university of Benin teaching Hospital, Decision-delivery of less than 15 minutes is required for cases of fetal hypoxia (cord prolapse, fetal distress, haemorrhage). Table 4 shows decision to delivery of five cases of fetal distress at University of Benin Teaching Hospital (UBTH).

**Table 4:** Case examples of spinal anaesthesia for fetal distress in UBTH

Patient	Fetal markers	Indication for spinal	Type of loading	Decision to delivery (Minutes)	APGAR scores (min)

1	Bradycardia, meconium stained liquor	Maternal refusal of GA	Co - loading	7	1^st^ 6/10 5^th^ 8/10
2	Reduced baseline variability	Maternal refusal of GA	Co - loading	10	1^st^ 5/10 5^th^ 6/10
3	Tachycardia, reduced baseline variability	Difficult airway,	Pre - loading	12	1^st^ 7/10 5^th^ 9/10
4	Cord proplapse, Bradycardia	Maternal refusal of GA	Co - loading	5	1^st^ 710 5^th^ 8/10
5	Late deceleration, absent fetal heart variability	Maternal preference	Pre - loading	6	1^st^ 5/10 5^th^ 7/10

### Increasing use of spinal anaesthesia for fetal distress: UBTH EXPERIENCE

Table 4 shows case examples of spinal anaesthesia for fetal distress at UBTH. Most mothers in this institution are afraid of death from general anaesthesia. They also prefer to be conscious to see the face and the sex of their babies. Review of anaesthetic techniques used for all Caesarean sections performed at UBTH between 2007 and 2011 revealed that more cases of Caesarean section due to fetal distress were performed under spinal anaesthesia at UBTH as shown in Table 5. In 2007, the incidence of spinal anaesthesia for fetal distress was 65.5% whereas it increased geometrically to 89% in 2011. Most of these Caesarean sections due to fetal distress were performed in less than 15 minutes.

**Table 5:** Review of anaesthesia for Caesarean section: UBTH (2007-2011)

parameter	2007	2008	2009	2010	2011

total no of C/S	878	1039	996	1321	1285
total no of Epidural	18	16	20	20	18
total no of GA for C/S	186	204	115	114	95
total no of Spinal Anaesthesia	674	819	861	1187	1172
total no of Fetal Distress	84	116	140	124	182
total no of GA for Fetal Distress	29	41	35	21	20
total no of Spinal for Fetal Distress	55	75	105	103	162
Percentage of Spinal Anaesthesia for Fetal Distress	65.5%	64.7%	75.0%	83.1%	89.0%

No, number; C/S, Caesarean section; GA, General anaesthesia.

Internationally, Obstetric anaesthesia protocol recommends regional anaesthesia over general anaesthesia for most Caesarean Section (15-17). The recommendation is based on the benefits of spinal anaesthesia as shown in Table 6. Algert *et al* (18) compared regional block versus general anaesthesia for Caesarean section and neonatal outcomes. They concluded that infants most affected by general anaesthesia were those already compromised in utero, as evidenced by fetal distress. They advised that the increased rate of adverse neonatal outcomes should be weighed up when general anaesthesia is under consideration for fetal distress (18). Marx *et al* (19) compared fetal neonatal status following Caesarean section for fetal distress and found that despite the presence of fetal distress, subarachnoid block was a most suitable method of anaesthesia in experienced hands. Sri *et al* (20) in their evaluation of anaesthesia methods in Caesarean section for fetal distress found that one and five-minute Apgar scores of the subarachnoid block group were significantly higher than those of the general anaesthesia group. One minute Apgar score of the ketamine group was significantly higher than that of thiopental group, but no significant difference in 5- minute Apgar score was found between the ketamine and the thiopental groups. They concluded that subarachnoid block is the choice of anaesthesia for fetal distress. General anaesthesia with ketamine Apgar score at one minute was better than that of the thiopental.

**Table 6:** Advantages of Spinal Anaesthesia

High patients satisfaction
Avoidance of aspiration pneumonitis
Avoidance of awareness
Patients’ preference
Patients discuss freely with care givers
Reduced incidence of PONV
Conscious mother, awake neonate
Reduced phase 1 recovery time
No instrumentation of airway
Avoidance of placental transfer of multiple drugs
Patients also monitor themselves
Less number of anaesthetists required
Low post operative morbidity
Immediate return to oral intake
Cost effectiveness

### How can you make your hands experienced?

You need to do as many simulated rapid spinal anaesthesia as possible in order to make your hands experienced. Before you can boast of performing rapid spinal anaesthesia for fetal distress, you must have rapidly performed many cases of spinal anaesthesia for both obstetric and non obstetric procedures. Your first time of performing rapid spinal anaesthesia should not be when you are faced with fetal distress. You must have been seen doing rapid spinal anaesthesia freely for other cases such as orthopaedic, gynaecological, and urological procedures. Practice is the key to your becoming an expert in rapid spinal anaesthesia. At UBTH, the competition is high. Most residents want to perform spinal anaesthesia in less than 2 minutes.

### Team Work: how ready are you?

Team work among the care givers is very crucial to rapid sequence spinal anaesthesia for fetal distress. Experienced obstetrician is as essential as experienced paediatrician and midwife. Every caregiver must be on standby in labour ward theatre. This helps in reducing unnecessary delay in mobilising care givers. Obstetric team are always willing to put up intravenous crystalloid right in the labour room using 16 G cannula. This helps to reduce time wasting to set up intravenous line in the operating room. Anaesthetists do assist paediatricians in resuscitating baby. Most of the time at UBTH, obstetricians do not wait for health assistants in order to transport the patient from labour ward to theatre. They throw job specification to the wind and transport the patients themselves. This makes decision-delivery time shorter.

### Fluid Management

Ordinarily, patients are supposed to be preloded with 1litre of crystalloid before induction of spinal anaesthesia but because of time constraint, this may not be possible. Co-loading is used in other to reduce time wasting in pre-loading. With placement of intravenous cannula, fluid loading with 1litre of crystalloid is carried out simultaneously while spinal anaesthesia is being induced. But spinal anaesthesia should not be performed before placement of intravenous cannula. Except for some situations that require fluid restriction, continuous crystalloid fluid (2-3 litres) should be given in order to mitigate the hypotension that may arise from sympathectomy using hyperbaric bupivacaine.

### Spinal technique

Patient is put in a sitting position. Some anaesthetists at UBTH sit up the patients with the two lower limbs on the operating table. This position is not convenient for the patients, this may be useful in non-obstetric patients. The proper sitting position with legs on the stool beside the operating table is desirable. L3L4 or L2L3 interspace is better than L4L5 interspace. Failure rate is higher with L4L5 interspace. Spinal pack must be on stand by in the operating room at all time. Preloading is possible during the waiting period for other care providers otherwise coloading is used. A single wipe of the back with chlorhexidine lotion is frequently used for scrubbing. Lidocaine infiltration is essential but can waste time in some instances. Lidocaine spray can be used in lieu of lidocaine infiltration whenever 25 G or bigger spinal needle is to be used. In case lidocaine infiltration or spray is to be avoided, smaller spinal needle such as 26 or 27 G can be used. Opioid as adjuvant to bupivacaine wastes a lot of time to constitute and measure, therefore, opioid should be avoided. Average of 2.2-2.5 ml of 0.5% hyperbaric bupivacaine is frequently used in our centres. Surgery starts almost immediately after cleaning and drapping of the patients by the obstetrician. Ephedrine is made handy and constituted in case there is hypotension which fluid alone cannot treat. Spinal pack is on standby in the operating suite waiting for the next Caesarean section. One touch, non touch spinal anaesthesia is desirable but second and third attempts can be helpful. The sequence for rapid spinal anaesthesia is outlined in Table 7. If the attending anaesthetist is unable to perform rapid spinal anaesthesia due to lack of expertise or there are delays in establishing the block, the plan should immediately change to general anaesthesia that is suitable for the patient.

**Table 7:** Nudgets for Rapid Spinal Anaesthesia

1. Standby experienced anaesthetists in labour ward theatre
2. Standby spinal pack in labour ward theatre
3. Pre-loading or co-loading is required with constituted ephedrine on standby
4. Intravenous line put in place in labour ward by obstetrician
5. No touch technique. Scrub and glove rapidly
6. Local infiltration is optional but helpful
7. One touch technique. Once the spinal needle has pierced the skin it must not be withdrawn until CSF is seen and drug is deposited. Difficulty in getting it may allow for the second touch
8. In the absence of loading, 5mg of ephedrine i.v is given prophylactically to prevent precipitous drop in blood pressure
9. Surgery starts almost immediately

## CONCLUSION

There is a pressing need for the strengthening of anaesthetists through training on modern techniques of subarachnoid block, especially registrar aneasthetists working in situations with limited resources. Infusion of strategies of rapid sequence spinal anaesthesia for fetal distress and introduction of rapid spinal anaesthesia protocol which involve maternal intravenous line access right in the labour room with continuous presence of spinal packs and care providers in labour ward theatre, among other strategies are necessary to popularise rapid sequence induction of spinal anaesthesia among world anaesthetists in general and African anaesthetists in particular. It is advisable to convert immediately to general anaesthesia whenever there is difficult spinal anaesthesia otherwise one should perform anaesthetic technique that is safe in one’s hands.
